# Editorial: Treatment outcomes, comorbidities and impact of discordant biochemical values in acromegaly

**DOI:** 10.3389/fendo.2024.1351350

**Published:** 2024-01-29

**Authors:** Claudia Campana, Eva Christine Coopmans, Sabrina Chiloiro

**Affiliations:** ^1^ Endocrinology Unit, Department of Internal Medicine and Medical Specialties, School of Medical and Pharmaceutical Sciences, University of Genova, Genova, Italy; ^2^ Division of Endocrinology, Department of Internal Medicine, Erasmus Medical Center, Rotterdam, Netherlands; ^3^ Division of Endocrinology, Department of Medicine, Leiden University Medical Center, Leiden, Netherlands; ^4^ Pituitary Unit, Fondazione Policlinico Universitario Agostino Gemelli, IRCCS, Rome, Italy; ^5^ Endocrinology and Diabetes Unit, Università Cattolica del Sacro Cuore, Rome, Italy

**Keywords:** acromegaly, neurosurgery, AIP, biochemical discordance, interventricular septum (IVS) thickness

Acromegaly is a rare disease characterized by the excess of growth hormone (GH) and subsequent increased secretion of insulin-like growth factor 1 (IGF-1) by the liver (1). In the vast majority the cases, the cause is a GH-secreting pituitary tumor (1). The hormonal excess leads to several comorbidities (e.g., cardiovascular, metabolic, and muscoloskeletal), impaired quality of life (QoL), and, if untreated, increased mortality (1, 2). To date, surgery is the first-line treatment for most patients, and the only therapeutic approach able to cure the patient, if complete resection is achieved (3). On this Research Topic, Vassileyva et al. performed a randomized controlled trial comparing microscopic and endoscopic surgery, showing a 1.4 times more frequent radical resection when using the endoscopic approach. Moreover, patients treated with endoscopic surgery had shorter post-operative hospital stay. However, despite the higher rate of complete macroscopical resection, there was no significant difference in disease remission rate at 12-month follow-up between endoscopic and microscopic procedure (72% and 68% of patients, respectively). Similarly, a previously published meta-analysis showed a slightly higher hormonal remission rate using the endoscopic approach (53.0% versus 46.7%) (4). Of note, it is of pivotal importance that the surgery is performed by an expert neurosurgeon, and the choice of the technique depends on the surgeon’s preference (3).

If patients refuse surgery, are not eligible or do not achieve surgical remission, medical treatment is indicated (3). The first line medical treatment is represented by first-generation somatostatin receptor ligands (fg-SRLs), achieving disease control in up to 55% of patients. In case of lack of response or complete resistance, the second generation somatostatin receptor ligand pasireotide or the GH receptor antagonist pegvisomant are indicated (3). Multiple clinical, radiological and molecular factors have been investigated in order to predict the response to medical therapy, in particular following treatment with fg-SRLs (5). Among these, the presence of aryl hydrocarbon receptor-interacting protein (AIP) mutation, or low AIP expression has been associated to poor fg-SRL response (6, 7). Trofimiuk-Müldner et al. investigated the prevalence of AIP variants in patients with apparently sporadic pituitary macroadenomas. In their cohort, including only adult patients, 3.8% of individuals had AIP variants. In this study, similarly to what previously reported, GH-secreting adenomas were the most common subtype presenting AIP variants. However, the AIP variants carriers did not differ substantially from patients with wild type gene; therefore, the routine screening of all patients with pituitary macroadenoma is not currently suggested.

The definition of disease control changed over time due to assays’ improvement, with the recently published last Consensus Statement on acromegaly recommending IGF-1 as the preferential biomarker, aiming to keep it in the middle-upper range of normality (8). Of note, the previous Consensus Statement recommended to monitor both GH and IGF-1 (3, 8). However, discrepancy between the two hormones, defined as normal age-adjusted IGF-1 and GH above a pre-defined cut-off (e.g., < 2.5 μg/L or <1 μg/L) or IGF-1 xULN (upper limit of normality) >1 and GH below the pre-defined cut-off, was present in up to 52% of patients with acromegaly (9–11). The impact of discordant GH and IGF-1 values on acromegaly-related comorbidities, QoL and mortality has not been fully elucidated, yet. However, the evaluation of “discordant” patients may be useful to further investigate the relative impact of GH and IGF-1 levels in acromegaly patients and their correlation with disease-related comorbidities. Romanisio et al. reported a real-life, single centre experience comparing patients achieving biochemical remission and patients with mildly discordant GH/IGF-1. In their cohort, the discrepancy between the two hormones did not lead to an increased risk of metabolic complications. Similarly, a previous study showed that diabetes mellitus and hypertension were not more severe in patients with discordant GH/IGF-1 compared to patients with normal IGF-1 and random GH<1 μg/L (12). Therefore, patients with mildly discordant GH and IGF-1 should not have a stricter follow-up compared to patients considered biochemically controlled for both parameters.

Of note, the specific role of GH and/or IGF-1 excess in the pathogenesis of the different acromegaly comorbidities is not completely understood. Cardiovascular disease is still an important cause of mortality in patients with acromegaly (2). Multiple factors are involved in the pathogenesis of the acromegaly cardiomyopathy, including both direct (presence of GH and IGF-1 receptors on cardiomyiocytes) and indirect effects (e.g., sodium retention, hypertension) (1).

Chen et al. investigated the possible relation between IGF-1 levels and interventricular septal (IVS) thickness in a cohort of 803 patients (including both individuals with and without acromegaly) for which a IGF-1 measurement was available. The Authors found a positive linear relation between IGF-1 levels and the IVS thickness. Of note, only 40 patients were affected by acromegaly and the results of the study did not change after their exclusion. In a subgroup analysis, high IGF-1 levels increased the risk of IVS thickening in males of all ages, and in female patients between 45- 60 years. Conversely, another study recently reported that, in patients with acromegaly, the left ventricular mass was correlated with GH levels but not with IGF-1 (13). Therefore, further studies are needed to investigate whether GH and IGF-1 exert a differential role on cardiac hypertrophy between patients with and without acromegaly.

In conclusion, the articles collected in this Research Topic highlight the complexity of acromegaly management, from the surgical approach, to the impact of GH/IGF-1 discordance on comorbidities. Furthermore, the knowledge derived from the investigation of acromegaly comorbidities could be useful to investigate the effect of GH and IGF-1 in subjects without acromegaly.

## Author contributions

CC: Writing – original draft. EC: Writing – review & editing. SC: Writing – review & editing.
